# Use of an HIV-risk screening tool to identify optimal candidates for PrEP scale-up among men who have sex with men in Toronto, Canada: disconnect between objective and subjective HIV risk

**DOI:** 10.7448/IAS.19.1.20777

**Published:** 2016-06-03

**Authors:** James Wilton, Taylor Kain, Shawn Fowler, Trevor A Hart, Troy Grennan, John Maxwell, Darrell HS Tan

**Affiliations:** 1 CATIE (Canadian AIDS Treatment Information Exchange), Toronto, Canada; 2 Department of Medicine, University of Toronto, Toronto, Canada; 3 Hassle Free Clinic, Toronto, Canada; 4 Department of Psychology, Ryerson University, Toronto, Canada; 5 Dalla Lana School of Public Health, University of Toronto, Toronto, Canada; 6 British Columbia Centre for Disease Control, Vancouver, Canada; 7 ACT (AIDS Committee of Toronto), Toronto, Canada; 8 Division of Infectious Diseases, St. Michael's Hospital, Toronto, Canada

**Keywords:** risk perception, risk behaviours, men who have sex with men, screening, pre-exposure prophylaxis, HIV

## Abstract

**Introduction:**

Identifying appropriate pre-exposure prophylaxis (PrEP) candidates is a challenge in planning for the safe and effective roll-out of this strategy. We explored the use of a validated HIV risk screening tool, HIV Incidence Risk Index for Men who have Sex with Men (HIRI-MSM), to identify “optimal” candidates among MSM testing at a busy sexual health clinic's community testing sites in Toronto, Canada.

**Methods:**

Between November 2014 and April 2015, we surveyed MSM undergoing anonymous HIV testing at community testing sites in Toronto, Canada, to quantify “optimal” candidates for scaling up PrEP roll-out, defined as being at high objective HIV risk (scoring ≥10 on the HIRI-MSM), perceiving oneself at moderate-to-high HIV risk and being willing to use PrEP. Cascades were constructed to identify barriers to broader PrEP uptake. The association between HIRI-MSM score and both willingness to use PrEP and perceived HIV risk were explored in separate multivariable logistic regression analyses.

**Results:**

Of 420 respondents, 64.4% were objectively at high risk, 52.5% were willing to use PrEP and 27.2% perceived themselves at moderate-to-high HIV risk. Only 16.4% were “optimal” candidates. Higher HIRI-MSM scores were positively associated with both willingness to use PrEP (aOR=1.7 per 10 score increase, 95%CI=1.3–2.2) and moderate-to-high perceived HIV risk (aOR=1.7 per 10 score increase, 95%CI=1.2–2.3). The proportion of men who were “optimal” candidates increased to 42.9% when the objective HIV risk cut-off was changed to top quartile of HIRI-MSM scores (≥26). In our full cascade, a very low proportion (5.3%) of MSM surveyed could potentially benefit from PrEP under current conditions. The greatest barrier in the cascade was low perception of HIV risk among high-risk men, but considerable numbers were also lost in downstream cascade steps. Of men at high objective HIV risk, 68.3% did not perceive themselves to be at moderate-to-high HIV risk, 23.6% were unaware of PrEP, 40.1% were not willing to use PrEP, 47.6% lacked a family physician with whom they felt comfortable discussing sexual health, and 31.6% had no means to cover the cost of PrEP.

**Conclusions:**

A higher HIRI-MSM cut-off may be helpful for identifying candidates for PrEP scale-up. Improving engagement in the PrEP cascade will require interventions to simultaneously address multiple barriers.

## Introduction

HIV infection rates are stable or rising among gay men and other men who have sex with men (MSM) in high-income countries, highlighting the limitations of current HIV control efforts and the need for new prevention strategies [1, 2]. HIV pre-exposure prophylaxis (PrEP) is a promising new approach recently found effective in multiple randomized placebo-controlled clinical trials [3–6]. Pharmacokinetic models suggest that the consistent use of PrEP may reduce the risk of HIV infection by up to 99% [7]. In 2012, the Food and Drug Administration (FDA) in the United States became the first regulatory agency to approve Truvada^®^ for HIV prevention [8]. This was followed by approvals in France, South Africa and Kenya in 2015, and Canada in 2016. Uptake of PrEP in the United States has been slow despite relatively early regulatory approval and widespread financial coverage [9] and has been even slower in other parts of the world, including Canada.

Identifying appropriate candidates for PrEP is an important challenge for scale-up of this strategy. As PrEP is expensive and carries potential risks related to side effects, renal and bone toxicity and HIV drug resistance, among others, there is consensus that it should be targeted towards those at highest HIV risk in order to maximize its clinical, public health and cost benefits [10–15]. To help identify and prioritize high-risk MSM for PrEP and other intensive HIV prevention interventions, the Centers for Disease Control and Prevention (CDC) in the United States published the HIV Incidence Risk Index for MSM (HIRI-MSM) HIV risk screening tool [16]. However, such “objective” assessments of high HIV risk should further be matched by a patient's subjective perception of being at elevated risk and willing to use PrEP, because both are likely to be important predictors of uptake and adherence [17–21]. Such strict prioritization is particularly important for initial scale-up in settings in which current PrEP access is limited, as many providers and clinics offering PrEP likely have limited capacity.

To plan for initial PrEP roll-out, we thus sought to determine the proportion of MSM testing for HIV at community outreach sites in Toronto, Canada who are at high objective HIV risk, have elevated perceived HIV risk, and are willing to use PrEP, as such individuals would be optimal candidates for linkage to PrEP providers. We further sought to identify personal and structural barriers to PrEP use among MSM at risk of HIV infection, to better understand what proportion could potentially benefit from this prevention strategy during future scale-up.

## Methods

Between November 2014 and April 2015, we distributed a 33-item anonymous paper survey among MSM presenting for anonymous point-of-care HIV testing at Hassle Free Clinic's satellite testing locations in the community. Hassle Free Clinic is a busy sexually transmitted infection (STI) clinic in downtown Toronto. Its satellite locations include two community health centres popular with the local gay community and an LGBTQ community centre. The clinic and its community satellites are potentially important sites for initial screening and linkage to PrEP services, as together they represent the largest anonymous rapid testing sites in Ontario (conducting approximately 5400 point-of-care HIV tests among MSM each year – with just over half performed at satellites) and has the highest positivity rate (1.7% among MSM) of all such sites in the province (Ken English and Shawn Fowler, personal communications, March 2016). However, the clinic and its satellite locations do not currently have the capacity to directly deliver PrEP. Basic demographics and HIV positivity rates are similar between MSM testing for HIV at the main clinic and its satellites. MSM who were able to understand English were eligible to complete the self-administered survey and did so prior to receiving HIV pretest counselling. All participants were offered a Can$5 gift card.

The survey covered demographics, sexual practices, recreational drug use, perceived HIV risk, awareness and usage of HIV PrEP and post-exposure prophylaxis (PEP), willingness to use PrEP and other biomedical prevention technologies, and condom use. Questions about PrEP were preceded by a short plain-language statement describing it as: “a new strategy for HIV prevention. It involves an HIV-negative person taking a pill DAILY, on an ongoing basis (starting before an exposure and continuing after for as long as the person is at risk) to reduce their risk of HIV infection. This pill contains two anti-HIV drugs (tenofovir/emtricitabine or Truvada^®^), and research suggests that it is generally safe and is over 90% effective if taken every day. It is much less effective if not taken every day and does not protect against other STIs. Taking PrEP would require a visit to a doctor every three months in order to be tested for HIV, STIs and side effects.” The survey was pilot tested by five participants for clarity and face validity, and the study was approved by Research Ethics Boards of the University of Toronto, Ryerson University, and St. Michael's Hospital prior to initiation.

Our primary objective was to estimate the proportion of participants who are “optimal” candidates for initial PrEP scale-up, defined as MSM meeting three criteria: high objective HIV risk, moderate-to-high perceived HIV risk, and willingness to use PrEP. High objective HIV risk was defined as scoring ≥10 on the HIRI-MSM, a validated 7-item screening tool for calculating HIV risk among MSM that incorporates age, number of male partners, number of HIV-positive male partners, frequency of condomless receptive anal sex, frequency of condomless insertive anal sex with HIV-positive partners and use of amyl nitrate (“poppers”) and methamphetamines [16]. Scores can range from 0 to 47 and scoring ≥10 is the suggested cut-off for prioritizing MSM for more intensive HIV prevention efforts such as PrEP, based on a sensitivity of 84% and a specificity of 45% for predicting incident HIV infection in the next six months.

Moderate-to-high perceived HIV risk was defined as answering “more than a little bit of risk” or “a lot of risk” on a four-point Likert-type scale in response to the question: “What do you think your current risk of getting HIV is?” Other response options included “no risk at all” and “a little bit of risk.” Willingness to use PrEP was defined as answering “agree” or “strongly agree” on a five-point Likert-type scale in response to the statement, “I would be interested in taking PrEP to reduce my current risk of HIV infection.” To gain further insight into willingness to use, our questionnaire included an open-ended question asking “What concerns do you have about PrEP?” Reponses were coded and reported for those not willing to use PrEP.

A secondary objective was to identify potential barriers to PrEP use by constructing a series of hypothetical “PrEP cascades”, as has been proposed elsewhere [22], analogous to the published concept of the HIV treatment cascade [23]. We reasoned that in order to benefit from PrEP, MSM must (A) be at risk of HIV infection, (B) be objectively at high risk, (C) perceive themselves to be at moderate-to-high risk, (D) be aware of PrEP, (E) be willing to use PrEP, (F) have a family doctor with whom they feel comfortable discussing their sexual health, and (G) have drug insurance coverage or be willing to pay the full cost of medications (Can$850/month) out-of-pocket. As all participants were seeking anonymous point-of-care HIV testing, all were considered part of population A. The primary outcome was thus defined by the cascade ABCE. To explore the relative importance of different cascade steps as barriers to engagement, we defined six other PrEP cascades as follows: ABCDEFG, ACDEFG, ABDEFG, ABCEFG, ABCDFG and ABCDEG, and analyzed the results visually for gaps.

Exploratory objectives were to quantify the relationship between HIRI-MSM score (predictor of primary interest) and both willingness to use PrEP (outcome #1) and moderate-to-high perceived HIV risk (outcome #2) in separate multivariable logistic regression analyses. After excluding variables due to collinearity, multivariable models were constructed using forward selection, with the HIRI-MSM score as the primary predictor of interest. Additional variables were considered one at a time and retained if statistically significant using a threshold of alpha=0.10. We assessed for collinearity using Spearman's correlation coefficients and chi-square tests for categorical variables. Where there was evidence for a strong correlation between two or more potential predictor variables (*p*<0.01) then only one was considered for inclusion in the multivariable models, based on model fit (AIC).

The target sample size was calculated based on the number of respondents needed to estimate the primary proportion of interest with reasonable precision using the equation $n=Z_{1-\alpha /2}^{2}$ *p(1−*p*)/*l* ^2^, where *Z*_1−α/2_ is the 1−α/2 critical value of the standard normal distribution, *p* is the proportion of interest and *l* is half the length of the desired 95% confidence interval [24]. Using a preliminary estimate of *p*=0.66 based on our prior study in which 66.1% of MSM described “definitely” or “maybe” being willing to use PrEP [25], and after allowing for 10% incomplete responses, we estimated that 387 or roughly 400 responses would be required.

## Results

Of the 1358 MSM testing for HIV at the community sites during the study period, a total of 420 (30.9%) participants completed the survey. Participant characteristics are described in Table 1. Median (interquartile range, IQR) age was 31 (26,38) years and most were white (55.2%), employed full-time (69.7%) and had a college/undergraduate education or higher (88.5%). More than half of men had drug insurance coverage (69.6%) and a family doctor with whom they felt comfortable discussing their sexual health (55.5%).

**Table 1:** Participant characteristics

Characteristic	
Age in years – median (interquartile range)	31 (26, 38)
Ethnicity – *n* (%)	
Caucasian	230 (55.2)
South Asian	26 (6.2)
Latino/Hispanic	43 (10.3)
Middle Eastern	24 (5.8)
Black	18 (3.8)
Asian (other)	60 (14.4)
Other^a^	18 (4.3)
Education – *n* (%)	
High school diploma or less	48 (11.5)
College/undergraduate degree	251 (60.3)
Professional or graduate degree	117 (28.1)
Employment – *n* (%)	
Full-time	290 (69.7)
Part-time	69 (16.6)
Unemployed	57 (13.7)
Drug use in past six months – *n* (%)	
Methamphetamines	32 (7.7)
Injectables	2 (0.5)
Alcohol	317 (76.4)
Crack cocaine	58 (14.0)
Poppers (Amyl nitrates)	123 (29.6)
Marijuana	178 (42.9)
Other recreational drugs	45 (10.8)
None	63 (15.2)
Lifetime diagnosis of STIs – *n* (%)	
Gonorrhoea	87 (20.9)
Chlamydia	70 (16.8)
Syphilis	27 (6.5)
Herpes	18 (4.3)
Genital warts	65 (15.6)

a Includes participants who indicated “Other” or more than one ethnicity.

A HIRI-MSM score could be calculated for 388 participants. The median (IQR) HIRI-MSM score was 15 (8,19), and the 90th percentile was 26. As such, most men (64.4%) scored ≥10 on the HIRI-MSM and therefore met the high objective HIV risk definition. Table 2 shows the contribution of the different HIRI-MSM variables to participants’ scores.

**Table 2:** Breakdown of HIRI-MSM and contribution of variables to individual scores

HIRI-MSM variable and response options	HIRI-MSM score	Number of participants (%)	Percent contribution to participants’ individual scores, Median (IQR)
1. Age			38.5% (26.3, 66.7)
< 18 years^a^ or ≥49 years	0	38 (9.2%)	
18–28 years	8	150 (36.1%)	
29–40 years	5	181 (43.6%)	
41–48 years	2	46 (11.1%)	
Total		415 (100%)	
2. Number of sex partners^b^			0% (0, 24.1)
> 10	7	63 (15.6%)	
6–10	4	108 (26.7%)	
0–5	0	234 (57.8%)	
Total		405 (100%)	
3. Condomless receptive anal sex with man of any status^b^			0% (0, 52.6)
1 or more times	10	183 (45.8%)	
0 times	0	217 (54.3%)	
Total		400 (100%)	
4. HIV-positive partners^b^			0% (0, 0)
> 1 positive partner	8	38 (9.4%)	
1 positive partner	4	46 (11.3%)	
< 1 positive partner	0	322 (79.3%)	
Total		406 (100%)	
5. Condomless insertive anal sex with HIV-positive man^b^			0% (0, 0)
5 or more times	6	9 (2.1%)	
0–4 times	0	411 (97.9%)	
Total		420 (100%)	
6. Methamphetamine use (crystal or speed)^b^			0% (0, 0)
Yes	5	32 (7.6%)	
No	0	388 (92.4%)	
Total		420 (100%)	
7. Popper use^b^			0% (0, 10.0)
Yes	3	123 (29.3%)	
No	0	297 (70.7%)	
Total		420 (100%)	

a Only 1 participant was <18 years

b In the past six months.

While most men were at objectively high HIV risk, only 27.2% (113/415) overall and 31.7% (79/249) of high-risk men perceived themselves to be at moderate-to-high HIV risk. Even among those in the highest quartile and decile of HIRI-MSM scores, only about half (43.6 and 54.3%, respectively) perceived themselves at moderate-to-high HIV risk. However, participants perceiving higher HIV risk were more likely to be objectively high risk compared to those perceiving no-to-low risk (78.2% vs. 59.4%, *p*<0.0001).

Slightly more than half of the respondents (52.5%, 214/408) indicated willingness to use PrEP. This proportion was greater among men at objectively high HIV risk (59.2%, 148/250) and among men who perceived themselves at elevated HIV risk (78.4%, 87/111). About half (50.3%) of the “high-risk” men who did not perceive themselves to be at moderate-to-high HIV risk were still willing to use PrEP. The most common concerns among men not willing to use PrEP were related to side effects (42.8%), risk compensation and potential increases in other STIs (14.4%), and cost (13.9%).

**Figure 1:**
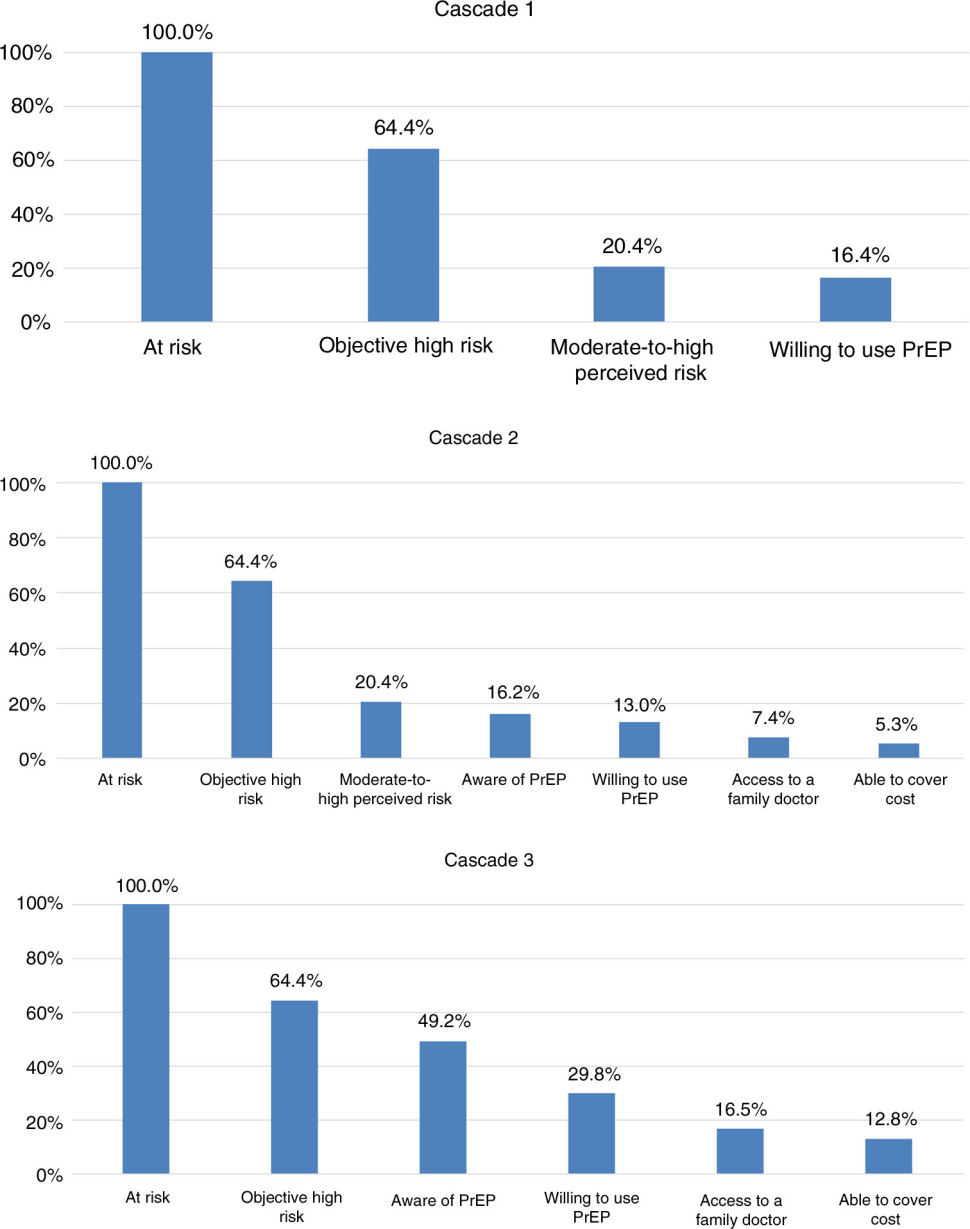
Proportion engaged in the PrEP cascade and potentially able to benefit from PrEP.

Only 16.4% (62/378) of participants met all three criteria (high objective HIV risk, moderate-to-high perceived HIV risk and willingness to use PrEP) and were thus considered “optimal” initial PrEP candidates (Figure 1, Cascade 1). Because of the high proportion of respondents who met the definition of objectively high HIV risk, we performed additional analyses in which objective high risk was redefined as the top decile of HIRI-MSM scores (i.e. ≥26), and found that the proportion meeting our definition of an “optimal” candidate was over 2.5-fold higher (42.9%, 15/35).

Awareness of PrEP was high at 72.0% and very similar to that of PEP (72.2%). Sources of PrEP awareness included the media (48.8%), a friend (39.0%), and healthcare providers (22.3%). Thirty-two respondents (7.9%) had ever used PEP and two (0.6%) reported PrEP use. The majority (76.3%) were unwilling to pay the full cost of PrEP out-of-pocket. However, 78.5% of the participants who were unwilling to pay the full cost were still willing to pay some of the monthly cost (median $100 Can, IQR=50, 160).

To inform future scale-up, potential barriers to PrEP use were explored in several additional cascades (Figure 1). According to cascade 2 – which included all proposed steps – a very low (5.3%, 20/377) proportion of MSM would be able to benefit from PrEP under current conditions. Cascades 1 and 2 demonstrate that the primary barrier is the large proportion of objectively high risk men who do not perceive themselves to be at moderate-to-high risk. Yet removing perception of HIV risk from the cascade only increased the proportion able to access PrEP to 12.8%, because considerable numbers of participants are still lost at every step in the cascade (Figure 1, Cascade 3). For instance, when descriptive analysis was limited to objectively high-risk participants (*n*=250), 68.3% did not perceive themselves to be at moderate-to-high risk, 23.6% were unaware of PrEP, 40.1% were not willing to use PrEP, 47.6% lacked a family physician with whom they felt comfortable discussing sexual health and 31.6% had no means to cover the cost.

Variables associated with willingness to use PrEP and moderate-to-high perceived HIV risk in exploratory logistic regression analyses are shown in Tables 3 and 4, respectively. The HIRI-MSM was associated with willingness to use PrEP in both univariable and multivariable models, with aOR=1.7 (95%CI=1.3, 2.2) per 10-point increase in score after adjustment for moderate-to-high perceived risk of HIV and prior use of PEP. Table 4 shows that the HIRI-MSM was also associated with moderate-to-high perceived HIV risk in both univariable and multivariable models (aOR=1.7, 95%CI=1.2, 2.3). Having less than a university education, being concerned about HIV risk and a prior history of a bacterial STI were also positively associated with elevated perceived HIV risk. In post-hoc univariable logistic regression analyses, we found that of the seven HIRI-MSM components, only age in the youngest (<18) or oldest (≥49) age categories (vs. 18–28 age category), ≥1 condomless receptive anal sex acts, ≥1 HIV-positive partners, and use of poppers had a significant positive association with higher perceived HIV risk (Table 5).

**Table 3:** Association between participant characteristics and willingness to use PrEP

	Univariable		Multivariable	
		
	OR (95% CI)	*p*	aOR (95% CI)	*p*
Age (by decade)	1.0 (0.8, 1.2)	0.7		
Ethnicity				
White	1.0			
Black	0.4 (0.1, 1.2)	0.1		
East Asian	0.8 (0.4, 1.4)	0.4		
Latino/Hispanic	1.5 (0.7, 2.9)	0.4		
Middle Eastern	1.2 (0.5, 2.7)	0.8		
South Asian	1.0 (0.5, 2.4)	0.9		
Other/mixed	1.1 (0.4, 2.9)	0.8		
Education				
College/university	1.0			
High school	2.4 (1.2, 4.6)	**0.01**		
Employed full time	1.0 (0.7, 1.4)	1.0		
Has and is comfortable with family doctor	0.9 (0.6, 1.3)	0.6		
Has private drug coverage	0.9 (0.6, 1.4)	0.7		
HIRI-MSM score (per 10 point increment)	1.8 (1.4, 2.4)	**<0.001**	1.7 (1.3, 2.2)	**<0.001**
Perceives moderate-to-high HIV risk	4.9 (3.0, 8.1)	**<0.001**	3.4 (2.0, 5.8)	**<0.001**
Concerned about HIV risk	4.2 (2.7, 6.4)	**<0.001**		
Aware of PEP	1.0 (0.7, 1.6)	1.0		
Prior use of PEP	3.6 (1.5, 8.5)	**0.004**	3.7 (1.4, 9.6)	**0.008**
Aware of PrEP	1.6 (1.0, 2.5)	**0.03**		
Heard about PrEP from healthcare provider	1.3 (0.7, 2.2)	0.4		
Heard about PrEP from friend	1.4 (0.8, 2.2)	0.2		
Heard about PrEP from media	1.1 (0.7, 1.8)	0.7		
Willingness to pay for PrEP out-of-pocket	2.4 (1.0, 5.6)	**0.04**		
Condom use				
Always	1.0			
Less than always	2.1 (1.3, 3.2)	**0.001**		
Speculated condom use if on PrEP				
No change	1.0			
Decreased condom use	1.5 (1.0, 2.3)	**0.04**		
Past history of bacterial STI^a^	2.0 (1.3, 3.0)	**<0.001**		
Past history of any STI^b^	2.0 (1.3, 3.0)	**0.002**		

a Bacterial STI=gonorrhoea, chlamydia or syphilis

b Any STI=gonorrhoea, chlamydia, syphilis, genital herpes or genital warts; €= constructed using forward selection, variables retained if alpha ≤ 0.1. Bold value indicates *p*<0.05.

**Table 4:** Association between participant characteristics and moderate-to-high perceived risk of HIV

	Univariable		Multivariable	
		
	OR (95% CI)	*p*	OR (95% CI)	*p*
Age (by decade)	1.2 (0.9, 1.4)	0.2		
Ethnicity				
White	1.0			
Black	0.9 (0.3, 2.8)	0.8		
East Asian	0.7 (0.4, 1.4)	0.4		
Latino/Hispanic	1.6 (0.8, 3.2)	0.2		
Middle Eastern	1.1 (0.4, 2.7)	0.9		
South Asian	0.5 (0.17, 1.5)	0.2		
Other/mixed	1.3 (0.5, 3.7)	0.6		
Education				
College/university	1.0		1.0	
High school	3.1 (1.7, 5.8)	**<0.001**	2.1 (1.0, 4.4)	0.05
Employed full time	1.3 (0.8, 2.2)	0.3		
Has and is comfortable with family doctor	0.7 (0.5, 1.1)	0.09		
Has private drug coverage	1.0 (0.6, 1.5)	0.9		
HIRI-MSM score (per 10 point increment)	2.1 (1.6, 2.7)	**<0.001**	1.7 (1.2, 2.3)	**0.001**
Concerned about HIV risk	8.4 (5.1, 13.7)	<**0.001**	7.3 (4.3, 12.5)	**<0.001**
Aware of PEP	1.0 (0.6, 1.7)	0.9		
Prior use of PEP	2.3 (1.1, 4.7)	**0.03**		
Aware of PrEP	1.3 (0.8, 2.2)	0.3		
Heard about PrEP from healthcare provider	1.5 (0.9, 2.7)	0.2		
Heard about PrEP from friend	1.1 (0.7, 1.8)	0.7		
Heard about PrEP from media	0.8 (0.5, 1.4)	0.5		
Willingness to pay for PrEP out-of-pocket	1.0 (0.4, 2.3)	1.0		
Condom use				
Always	1.0			
Less than always	4.7 (2.5, 8.9)	**<0.001**		
Speculated condom use if on PrEP				
No change	1.0			
Decreased condom use	1.0 (0.6, 1.6)	1.0		
Past history of bacterial STI^a^	2.2 (1.4, 3.5)	**<0.001**	2.1 (1.2, 3.7)	**0.009**
Past history of any STI^b^	2.1 (1.7, 3.3)	**<0.001**		

a Bacterial STI=gonorrhoea, chlamydia or syphilis

b Any STI=gonorrhoea, chlamydia, syphilis, genital herpes or genital warts; €= constructed using forward selection, variables retained if alpha ≤ 0.1. Bold value indicates *p*<0.05.

**Table 5:** Association between HIRI-MSM components and moderate-to-high perceived HIV risk

	Number (%) reporting moderate/high perceived HIV risk	Univariable models	
	
		OR (95% CI)	*p*
Age			
18–28 years	34/149 (22.8%)	1.0	
29–40 years	51/179 (28.5%)	1.6 (0.9–2.9)	0.2
41–48 years	10/46 (21.7%)	0.8 (0.3–2.2)	0.7
< 18 years^a^ or 49 years or more	16/38 (42.1%)	4.1 (1.7–9.8)	**0.002**
Number of sex partners^b^			
0–5	54/234 (23.1%)	1.0	
6–10	30/108 (27.8%)	0.7 (0.4–1.3)	0.2
> 10	24/62 (38.7%)	0.7 (0.3–1.6)	0.4
Condomless receptive anal sex^b^			
0 times	39/219 (18.1%)	1.0	
1 or more times	69/183 (37.7%)	3.2 (1.9–5.3)	<**0.0001**
HIV-positive partners^b^			
0	67/321 (20.9%)	1.0	
1	18/46 (39.1%)	2.8 (1.4–5.8)	**0.005**
> 1	21/38 (55.3%)	3.1 (1.3–7.7)	**0.01**
Condomless insertive anal sex with HIV-positive partner^b^			
0–4 times	106/406 (26.1%)	1.0	
5 or more times	7/9 (77.8%)	5.7 (0.9–35.6)	0.06
Popper use^b^			
No	62/292 (21.2%)	1.0	
Yes	51/123 (41.5%)	2.4 (1.3–4.4)	**0.004**
Methamphetamine use^b^			
No	97/383 (25.3%)	1.0	
Yes	16/32 (50.0%)	1.2 (0.5–3.2)	0.7

a Only 1 participant was <18 years

b In the past six months. Bold value indicates *p*<0.05.

## Discussion

Targeting PrEP to those at highest risk is important to limit its potential risks (side effects, toxicity, risk compensation) and maximize cost-effectiveness. Current guidelines recommend that PrEP be considered for those “at substantial risk of HIV acquisition” [12], but evidence-based guidance on how to conduct this assessment is scarce [26]. In this study, we applied a validated MSM-specific HIV risk screening tool to MSM seeking anonymous point-of-care HIV testing [16] and found that the majority (64.4%) met the recommended high-risk cut-off score of ≥10. To our knowledge, this is the first quantitative study to prospectively explore use of the HIRI-MSM for its suggested purpose.

We also considered perception of HIV risk and willingness to use PrEP, reasoning that high-risk MSM who also meet these criteria would be an optimal population to target for early PrEP uptake. Indeed, higher perceived HIV risk has been associated with greater uptake and adherence in double-blinded and open-label PrEP studies [17–21]. We found that many objectively high-risk men did not perceive themselves to be at moderate-to-high HIV risk (68.3%) or were not willing to use PrEP (40.1%), leaving only 16.4% as “optimal” initial candidates for PrEP. The discordance between objective HIV risk and the other two criteria (subjective HIV risk and willingness to use) highlights an important challenge when assessing individuals for PrEP. One potential reason for this disparity may be that the HIRI-MSM score cut-off for defining objective “high-risk” was too low. Using a higher cut-off may be a practical way of prioritizing MSM for more intensive PrEP assessments, particularly for the initial scale-up of PrEP in busy clinical contexts such as ours. Promisingly, higher HIRI-MSM scores were associated with greater perception of HIV risk and willingness to use PrEP, and the proportion of men meeting all three criteria jumped to 42.9% when objective HIV risk was defined as HIRI ≥ 26. Data from which the HIRI-MSM was derived and validated show that a cut-off of ≥26 corresponded to a specificity of 91.6–93.0% for incident HIV infection in the next six months [16]. On the other hand, using a higher cut-off may limit the public health impact of PrEP and therefore selection of an appropriate threshold should consider the availability of time, resources and capacity of local PrEP services.

Low perception of HIV risk among “high-risk” MSM has been documented in other studies [27–30]. Many men may simply not recognize the HIV risk associated with some characteristics within the HIRI-MSM scoring system, despite their objective relationship with incident HIV [31]. Studies suggest perception of HIV risk among gay men can be influenced by factors not included in the HIRI-MSM, such as type of sex partners (casual/main), perceived monogamy, and other sex partner characteristics [32, 33]. Further investigation suggested that participants in our study recognized the HIV risk associated with most components within the HIRI-MSM. However, younger men were not more likely perceive higher HIV risk, yet this component (i.e. younger age) contributed the most to participant's HIRI-MSM scores. When screening MSM for PrEP eligibility, perception of HIV risk should be explored and misconceptions about HIV transmission addressed, particularly among younger MSM. That popper use was associated with higher perceived HIV risk, and also the fourth most important contributor to HIRI-MSM scores suggests it may be an important factor for identifying optimal PrEP candidates. Further research is needed to understand disparities between objective and subjective HIV risk and evaluate strategies to bridge this gap. Of note, an ongoing randomized controlled study is evaluating whether informing a patient of his/her HIV risk score (obtained using a modified HIRI-MSM tool) can increase the likelihood of PrEP uptake [34].

In our study where PrEP awareness was high (72.0%), willingness to use PrEP was also relatively high (52.5%) and comparable to that reported in other surveys among MSM (reviewed in [35]). Safety (42.8%) was the most common concern expressed by participants who were not willing to use PrEP. This has been identified as the most common reason for not initiating PrEP in open-label studies [18–21], even though the daily use of TDF/FTC as PrEP appears to be safe and well tolerated. Educational resources and campaigns promoting PrEP uptake should emphasize its favourable safety profile.

Interestingly, 50.3% of “high-risk” men who did not perceive themselves to be at moderate-to-high HIV risk were still willing to use PrEP, suggesting alternate motivations for wanting to use PrEP that could be harnessed to optimize PrEP uptake. For instance, a recent study of HIV-negative MSM in primary partnerships found that intimacy motivations for condomless sex – but not higher HIV risk perceptions – were independently associated with PrEP adoption intentions in multivariable analysis [36]. It may thus be useful for providers to explore this motivation with clients who are uncertain of their primary partner's HIV status or risk behaviours. In addition, several qualitative studies suggest that PrEP may decrease anxiety about sex [37, 38]. Such psychological benefits could be attractive for many at-risk individuals contemplating PrEP, given the high burden of syndemic mental health issues in such populations, although further work is needed to quantify broader impacts on mental health and function. Finally, PrEP is also seen as a way to increase sexual pleasure [32]. To the extent that all these motivations may reflect intentions to decrease existing levels of consistent condom use in some individuals, the emergence of PrEP challenges front-line providers to have increasingly open and honest conversations with patients about balancing desirable and undesirable outcomes related to sexual behaviour.

In our study, every 10-point increase in the HIRI-MSM increased the odds of willingness to use PrEP by 70%. Other studies among MSM have also found higher risk sexual behaviours (such as condomless sex) to be associated with hypothetical interest in using PrEP [28, 29] [35], as well as actual uptake in open-label studies [20, 21]. Other characteristics independently associated with willingness to use PrEP in our study included higher perceived HIV risk and prior use of PEP, the latter finding highlighting the potential to transition individuals from PEP to PrEP [39, 40]. Further work is needed to better understand the relative preferences regarding the use of new HIV prevention technologies among MSM, for instance, by using discrete choice experiments [41, 42].

The concept of a “PrEP Cascade” has been proposed to understand the individual and structural factors which may prevent an HIV-negative individual benefitting from PrEP and undermine its potential public health impact [22]. In our full hypothetical cascade including all proposed steps, only 5.3% of the participants would potentially be able to benefit from PrEP. While low perceived HIV risk was the largest “leakage” point in this cascade, removing perception of risk only increased the proportion able to benefit to 12.8%, highlighting the importance of simultaneously addressing other downstream steps, including PrEP awareness, the availability of suitable PrEP providers, willingness to use PrEP, and drug cost coverage to improve engagement. Another study of a hypothetical PrEP cascade with similar steps as ours (but without HIRI-MSM or perception of HIV risk) estimated that only 15.2% of MSM in Atlanta, Georgia, would achieve protection from PrEP [43]. Hence, it is not surprising that PrEP use in our Toronto-based study was limited to only two participants. Collection of empirical data from demonstration projects is needed to develop a more complete PrEP cascade and to better understand “real-life” barriers.

Our strategy for screening MSM had several potential limitations. We used single items for assessing willingness to use PrEP and perceived HIV risk. However, we felt that they were adequate for initial screening in order to facilitate linkage to dedicated PrEP services where more comprehensive assessments could be conducted on a case-by-case basis. Such assessments should consider other important factors, including sexual pleasure [44], self-efficacy to use condoms [45], stigma and discrimination, and freedom of choice. Also, our definition of optimal PrEP candidacy has not been validated for prediction of actual PrEP uptake and adherence. Several limitations of our study findings are related to the HIRI-MSM tool itself. First, the HIRI-MSM was developed for use among MSM in the United States, and its validity as an objective measure of HIV risk has not been evaluated in our setting. Second, the HIRI-MSM is intended as a screening tool to be used in conjunction with clinical judgement for identifying candidates for intensified HIV prevention efforts, not a definitive decision-making tool. However, underestimation of HIV risk was seen even in those with the highest objective risk scores.

This study had a number of other limitations. The survey was self-administered and only included basic information about PrEP, rendering some findings subject to hypothetical bias (wherein respondent reports of what they think they would do may differ from what they would actually do) because they did not require real action on the part of respondents [46]. However, “willingness to use” has emerged as a common measure of acceptability for emerging HIV prevention technologies [35] and may be helpful in planning for real-world implementation as was the intent in our study. Our sample included a disproportionate number of fully employed, highly educated MSM, limiting the generalizability of our findings to socio-economically disadvantaged MSM. Nevertheless, anonymous HIV-testing facilities such as our recruitment sites represent a potentially important site for identifying potential PrEP candidates. Furthermore, we were unable to measure potential selection bias as it was not feasible to collect data on those who did not complete the survey. While the response rate was relatively low, it is important to note that not all MSM testing for HIV during the study period were approached for study participation. Finally, we recognize that HIV risk is not static [47], and our study only captured information on HIV risk at a specific time. In people using PrEP, it is important to monitor HIV risk on a continuous basis.

## Conclusions

PrEP represents an important opportunity to improve HIV prevention for gay and other MSM. This study is the first to use a validated HIV risk screening tool to prospectively determine PrEP eligibility among MSM. Our results suggest a higher cut-off than what is proposed by the tool's authors may be more appropriate for identifying PrEP eligible men in terms of feasibility and efficiency. We also identified several personal and structural barriers to PrEP uptake among eligible men, particularly the large number of objectively high-risk men who did not perceive themselves at elevated HIV risk. Further work is needed to optimize strategies for determining PrEP eligibility, bridging the disconnect between objective and subjective HIV risk, and improving engagement in the PrEP cascade.
